# Introduction to the *RSC Advances* Emerging Investigators Series 2022

**DOI:** 10.1039/d3ra90054a

**Published:** 2023-06-22

**Authors:** Fabienne Dumoulin, Shirley Nakagaki

**Affiliations:** a Acibadem Mehmet Ali Aydinlar University Turkey; b Universidade Federal do Paraná Brazil

## Abstract

Dr Fabienne Dumoulin and Professor Shirley Nakagaki are delighted to introduce the *RSC Advances* Emerging Investigators Series, which highlights some of the very best work of early career researchers.
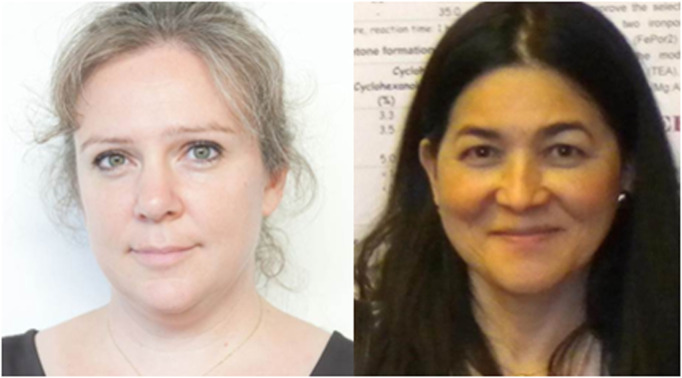

We are delighted to present the 2022 edition of the *RSC Advances* Emerging Investigators Series. Following the success of our inaugural 2021 edition, which featured 23 exceptional works from diverse research fields and countries, we are excited to continue highlighting the chemistry research being conducted by some of the leading investigators in our community. These promising researchers were selected from the recommendations of our Editorial Board members and Associate Editors. Authors can also self-nominate for participation in this series, which showcases the impressive breadth and caliber of innovative and impactful work of emerging investigators in chemistry.

The 2022 edition of the *RSC Advances* Emerging Investigators series reinforces our commitment to supporting emerging investigators who will play a key role in shaping the future of chemistry in their respective countries. We extend our sincere gratitude to all the authors for their exceptional contributions, as well as to the editors and referees for their collaboration, which has resulted in this high-quality series.

The articles featured come from various countries and continents, delving into molecular and solid-state chemistry with diverse applications, primarily in the areas of green and environmental chemistry, as well as biological and bioinorganic chemistry. Additionally, we have included papers that propose theoretical calculations as solutions to chemistry problems.

We start the 2022 series with an article by Vaz *et al.* from the University of Aveiro, Portugal (https://doi.org/10.1039/d2ra02581g), which discusses the recovery of bacterioruberin and proteins from *Haloferax mediterranei* using tensioactive solvents. This recovery is important because marine resources, such as algae and cyanobacteria, have gained significant interest as renewable feedstocks to produce several natural bioactive compounds. Haloarchaea microorganisms are little-explored marine resources that have the potential to be a promising source of valuable compounds, such as carotenoids (including beta-carotene, salinixanthin, bacterioruberin, and its precursors lycopene and phytoene), with unique characteristics due to their adaptation to extreme environments like extreme saline conditions.

We then go to a study by James *et al.* from Cochin University of Science and Technology and University of Calicut, India (https://doi.org/10.1039/d2ra03394a). In this article, the authors present molecular dynamics simulations coupled with quantum mechanical studies of hydrolases (such as cutinase) in the context of enzymatic processes aimed at accelerating the hydrolysis of polyethylene terephthalate (PET).

Next, Hancock *et al.* from the University of Melbourne, Australia (https://doi.org/10.1039/d2ra01703b) report on the binding of four different aromatic excimer models, analyzed through dissociation curves. They claim that this is the first study to provide single-reference wave function curves at the complete basis set (CBS) limit for aromatic excimer systems.

He *et al.* from Lehigh University and Procter & Gamble Co., USA (https://doi.org/10.1039/d2ra00609j), focus on the characterization of rheological properties for consumer, fabric, and home care products using rheological modifiers. By providing a guide to determine the necessary composition for desired rheological properties, this article eliminates the need for trial-and-error experiments during product formulation. The goal of the study was to gain a better understanding of the effect of each component concentration on the rheological properties of a formulation. This multi-center research sheds light on the importance of studying rheological properties in developing effective product formulations.

The fifth article comes from Marçon *et al.* from the University of Campinas, Brazil (https://doi.org/10.1039/d2ra00588c) and focuses on catalysis and green chemistry. The authors describe the flow synthesis of furfuryl alcohol and DHMF (dihydroxymethylfuran) from their respective aldehydes, using a Meerwein–Ponndorf–Verley reaction (MPV) with iso-propanol catalysed by basic zirconium carbonate. The study aims to develop new strategies for synthesizing bio-based compounds using a continuous flow process, which provides opportunities for greener processes. The authors also emphasize the pivotal role of using an inline FT-IR device for continuous monitoring, which allowed for fast process optimization.

The field of catalysis has another interesting contribution, but with a focus on bioinorganic studies. Mouli *et al.* from the Indian Institute of Technology-Hyderabad, Kandi, India (https://doi.org/10.1039/d1ra06558k) prepared and investigated a metal flavin model complex as a catalyst for aerobic sulphoxidation. Their goal was to develop bio-inspired compounds that mimic the activity of certain flavoenzymes that exhibit monooxygenase activity.

Next, we have a fascinating study that explores strategies for improving the permeability of synthetic BBB shuttle peptides, specifically angiopep-2 (Ang2), and monoclonal antibody (mAb) compounds, across the blood–brain barrier. This article has significant implications for antibody-based therapy for central nervous system (CNS) diseases, as it offers a promising approach to enhancing the therapeutic efficacy of antibody agents. Anami *et al.* from the University of Texas Health Center at Houston, USA (https://doi.org/10.1039/d1ra08131d) conducted this work and their findings have exciting potential for advancing treatments for neurological disorders.

The eighth work, conducted by do Nascimento *et al.* from different research groups at the Federal University of Rio de Janeiro, Brazil, and University Artois, France (https://doi.org/10.1039/d1ra08111j), focuses on the utilization of abundant lignocellulosic biomass residues in Brazil. The authors report on the acylation of an anhydrous carbohydrate, the most abundant organic substance present in biomass, to obtain carbohydrate fatty-acid esters *via* enzymatic acylation of levoglucosan. Lipase B from *Candida antarctica* (CALB) was immobilized in epoxy resin with different acyl donors in continuous flow. The obtained compounds were evaluated as antibacterial agents against *Staphylococcus aureus*, demonstrating promising results in comparison to other similar compounds reported in the literature. This article highlights the potential of using lignocellulosic biomass residues as a source of valuable compounds for various applications.

An interesting study on water remediation and purification was conducted by Stern *et al.* from Louisiana State University in Baton Rouge, USA (https://doi.org/10.1039/d2ra05943f). The contamination of water with oxyanion chromium(iv) from various industrial activities, such as leather tanning, chrome plating, dye and pigment manufacturing, and others, is a serious environmental issue due to its high toxicity. Cr^6+^ can enter cells through the sulfate uptake pathway, making it particularly harmful. The toxicity of Cr^3+^ is much lower, and this study aimed to promote the electrochemical reduction of Cr^6+^ to Cr^3+^ using glassy carbon electrodes under optimal pH, concentration, and buffer conditions. An important finding of this study was that the chemical structure of the buffer impacted the electrochemical process. The electrochemical method appears to be a promising approach for removing this metal-ion contaminant from drinking water, as it minimizes the waste produced.

In a similar vein, the tenth and final article by Silva do Nascimento *et al.* from Universidad Nacional del Sur, Bahia Blanca, Argentina (https://doi.org/10.1039/d2ra07405b) investigates a solid composed of montmorillonite encapsulated in an alginate hydrogel composite for pollutant adsorption purposes. The authors focus on the family of surfactant pollutants, which are frequently discharged into the environment, with water-soluble cationic surfactants being particularly effective as antimicrobial agents. One of the most popular antiseptics and disinfectants in the world, BAC (benzalkonium chloride), is also a common ingredient in disinfectants recommended for use as a prophylaxis protocol for viruses such as SARS-CoV-2. The authors demonstrate that the montmorillonite/alginate composite can adsorb cationic surfactants, and X-ray diffraction (XRD) is used to elucidate the surface reactivity of the clay encapsulated in the alginate hydrogel bead, thus highlighting the adsorption process of cationic pollutants within the composite.

We hope that this second edition of the *RSC Advances* Emerging Investigators Series will continue to inspire all our readers and motivate new scientists around the world to pursue research in the highlighted fields and beyond. We invite them to join us in future editions of this series and be a part of shaping the future of scientific innovation.

## Supplementary Material

